# The Role of Nitrate-Reducing Bacteria Isolated from *Helicobacter pylori*-Infected Individuals in Gastric Cancer Development

**DOI:** 10.3390/microorganisms14040760

**Published:** 2026-03-27

**Authors:** Serika Kuwagi, Kazuyoshi Gotoh, Marina Komatsubara, Shuma Tsuji, Shyoutarou Okanoue, Hiroyuki Okada, Jumpei Uchiyama, Akari Watanabe, Kenji Yokota

**Affiliations:** 1 Department of Bacteriology, Academic Field of Health Sciences, Okayama University, Okayama 700-8558, Japan; pj7k60rw@s.okayama-u.ac.jp (S.K.); gotok@okayama-u.ac.jp (K.G.); komatsumari@s.okayama-u.ac.jp (M.K.); tsuji-s@s.okayama-u.ac.jp (S.T.); 2 Department of Gastroenterology and Hepatology, Academic Field of Medicine Dentistry and Pharmaceutical Sciences, Okayama University, Okayama 700-8558, Japan; shotaro.bc.19@gmail.com; 3 Himeji Red Cross Hospital, Himeji 670-8540, Japan; hiro@md.okayama-u.ac.jp; 4 Department of Bacteriology, Academic Field of Medicine Dentistry and Pharmaceutical Sciences, Okayama University, Okayama 700-8558, Japan; uchiyama@okayama-u.ac.jp; 5 Department of Oral Health Care and Rehabilitation, Institute of Biomedical Sciences, Graduate School, Tokushima University, Tokushima 770-0042, Japan; akari.watanabe@tokushima-u.ac.jp

**Keywords:** *Helicobacter pylori* infection, gastric cancer, nitrate-reducing bacteria, gastritis

## Abstract

*Helicobacter pylori* is a Gram-negative bacterium that inhabits the gastric mucosa, with a global prevalence in humans of approximately 40%. It is likely the cause of 90% of gastric cancer (GC) cases and thus considered the most prominent driver of GC development. However, during gastric mucosal atrophy, other bacteria such as nitrate-reducing bacteria (NRB) also proliferate. In this study, we isolated NRB from patients with gastritis and GC to examine their effects on the epithelial cell cycle and production of various cytokines in monocytic cell lines. Bacterial counts (excluding *H. pylori* and NRB) increased with the progression of gastric mucosal atrophy and were significantly higher in patients with GC. Gastric epithelial cell lines were stimulated with isolated NRB, and the proportion of cells in each cell cycle was measured. Strains from patients with open-type gastritis progressed more rapidly through cell cycles than those from patients with GC. NRB isolated from gastric cancer had high nitrate-reducing activity. Thus, NRB may contribute to GC progression during *H. pylori*-induced carcinogenesis. Therefore, evaluating gastric atrophy and microbiota may be important for managing the risk of GC.

## 1. Introduction

*Helicobacter pylori* is a Gram-negative bacterium that inhabits the gastric mucosa, with a global prevalence in humans of approximately 40% [1,2]. *H. pylori* is considered to be responsible for 90% of gastric cancer (GC) cases and is widely recognized as the most important driver of GC development [3]. In Japan, *H. pylori* eradication therapy was approved for coverage by health insurance companies in 2013. Since then, the number of deaths from GC has decreased due to a decrease in the number of *H. pylori* infections [4]. This clearly demonstrates the association between *H. pylori* infection and GC. However, because only 2.9% of patients with *H. pylori* infection develop GC [5], other factors may be involved in *H. pylori*-induced GC.

*H. pylori* infection can promote the progression of atrophic gastritis, which in turn decreases gastric acid secretion and increases gastric pH, allowing bacteria other than *H. pylori* to proliferate in the stomach. These other gastric microbiota may also be involved in the development of GC. For example, in one study, INS-GAS mice with complex gastric microbiota developed gastric intraepithelial neoplasia seven months after *H. pylori* infection; however, the tumor incidence rate in INS-GAS male mice infected with *H. pylori* alone remained at 44.4%, even 11 months after the *H. pylori* infection [6]. Another study showed that co-infection with *H. pylori* and three mutant Schaedler’s microbiota or various other gut microbiota promoted the progression of gastric lesions and intraepithelial neoplasia [7].

Among the bacteria constituting the gastric microbiome, nitrate-reducing bacteria (NRB) are thought to be involved in GC development. NRB reduce nitrates to produce nitrites and react with secondary amines to produce carcinogenic N-nitroso compounds. Although N-nitroso compounds may be introduced through exogenous sources such as diet and cigarette smoke, endogenous formation is believed to be more prevalent [8]. The European Prospective Investigation into Cancer and Nutrition showed that the index of endogenous nitroso compound (ENOC) formation correlated with GC risk. Furthermore, ENOC formation was significantly associated with GC risk in *H. pylori*-infected individuals, whereas dietary nitrosodimethylamine was not [9]. Therefore, the proliferation of NRB in the stomach and the resulting increase in the production of N-nitroso compounds may increase the risk of GC development.

Previous studies have frequently isolated bacteria such as *Acinetobacter*, *Fusobacterium, Lactococcus*, *Lactobacillus*, *Peptostreptococcus*, *Prevotella*, *Streptococcus*, *Selenomonas*, and *Veillonella* from the stomachs of patients with GC [10], whereas other studies have shown that *Streptococcus* is the most common bacterium found in the gut microbiota of patients with chronic gastritis [11]. Furthermore, a study examining the gastric microbiota of patients with gastritis and GC calculated a dysbiosis index based on data from the most relevant genera characterizing each patient group; this improved the sensitivity and specificity of GC detection compared to detection using only one genus. This suggests that changes in the bacterial community rather than in individual taxa contribute to the development of GC [11].

Previous studies have not revealed whether a specific composition of the gastric microbiome is a risk factor for GC, or whether the diversity of gastric microbiota affects GC development. Furthermore, most of these studies involved genetic analysis of patients’ gastric microbiota but did not investigate the effects of the bacteria on gastric cells.

We have previously shown that co-culture stimulation with NRB and *H. pylori* from patients with gastritis strongly induces cytotoxicity and acute inflammatory responses and that NRB promote cell cycle progression [12]. In this study, we isolated numerous NRB species and strains from patients with gastritis or GC. We investigated whether NRB are a risk factor for GC development by examining their effects on gastric cells.

## 2. Materials and Methods

### 2.1. Isolation and Identification of Nitrate-Reducing Bacteria

#### 2.1.1. Patients

*H. pylori* cultures were obtained from mucosal biopsies taken from the greater curvature of the antrum and gastric body during upper gastrointestinal endoscopy in 91 patients with gastritis (61 patients with mild, closed-type gastric mucosal atrophy and 30 patients with advanced, open-type gastric mucosal atrophy) caused by *H. pylori* infection and 31 patients with GC. Clinical information (patient information and progression of gastric mucosal atrophy according to Kimura and Takemoto’s endoscopic classification) was obtained to evaluate the progression of atrophic gastritis based on the planar spread of the gastric mucosa and to classify the atrophic gastritis either as a closed type (C-1 to C-3) or an open type (O-1 to O-3). In closed-type atrophic gastritis, the endoscopic atrophic border is on the lesser curvature side of the gastric body and does not extend beyond the cardia, whereas in open-type atrophic gastritis, the border extends beyond the cardia and toward the greater curvature. All patients provided informed consent before biopsies. This study was approved by the Clinical Research Ethics Committee (No. K1903-025) of Okayama University.

#### 2.1.2. Culture and Identification of Bacterial Species

Bacteria other than *H. pylori* were identified by 16S rDNA sequencing [12], and the isolated bacteria were subcultured and evaluated for their nitrate-reduction ability.

#### 2.1.3. Bacterial Culture

Gastric biopsy specimens were homogenized, smeared on BHI medium supplemented with 7% horse blood and cultured for one week. After culturing, *H. pylori* was identified, and the resulting bacteria were subcultured on BHI medium for two to three days.

### 2.2. Nitrate-Reduction Activity Measurements

#### 2.2.1. Subjects

Twenty-six NRB strains isolated from 18 patients with gastritis or GC were analyzed and divided into fast- and slow-growing strains (Table 1).

#### 2.2.2. Cultivation and Measurement Procedures

Each NRB strain (Table 1) was inoculated into potassium-nitrate-supplemented broth. Fast-growing strains were cultured in an aerobic incubator, and slow-growing strains were cultured in a microaerobic incubator, both at 37 °C. Nitrite and bacterial masses were measured over time (fast-growing strains: 1, 3, 6, 12, 24, 48, 72, and 96 h after inoculation; slow-growing strains: 3, 6, 24, 48, 72, and 96 h after inoculation).

#### 2.2.3. Nitrite Concentration Measurement

Potassium nitrite and sodium nitrite were dissolved in nitrate-supplemented broth to prepare a dilution series (1000, 500, 250, 125, 62.5, 31.2, 15.6, and 7.8 mg/mL). An amount of 2.5 µL each of α-naphthylamine acetate and sulfanilic acid solutions were added to the prepared potassium nitrite and sodium nitrite solutions and thoroughly mixed. A positive result will produce a red color, and absorbance was measured at a wavelength (405 nm), which approximately absorbs red light. Absorbance measurements were performed by adding 200 µL of nitrite solution to a 96-well plate and measuring the absorbance using a MULTISKAN FC instrument (Thermo Fisher Scientific, Waltham, MA, USA). A calibration curve was then created.

A total of 1 mL of the cultured bacterial solution was placed in a 1.5 mL tube and centrifuged at 15,000 rpm for 10 min. After centrifugation, the supernatant was transferred to a separate tube, and 12.5 µL each of α-naphthylamine acetate and sulfanilic acid solutions were added and mixed thoroughly. The resulting solution was subsequently placed in a 96-well plate (200 µL in each well), and the absorbance was measured at 405 nm using a MULTISKAN FC instrument (Thermo Fisher Scientific, Waltham, MA, USA). This absorbance value was used as a direct indicator of the nitrite concentration.

#### 2.2.4. Creation of Bacterial Growth Curve

The cultured bacterial solution was placed in a 1 mL cell, and the absorbance was measured at 600 nm. The absorbance value was used as a direct indicator of bacterial mass.

### 2.3. Effects of Bacterial Stimulation on the Cell Cycle

#### 2.3.1. Subjects

We selected bacteria with a high nitrate-reducing ability. Twenty-six bacterial strains were isolated from the 18 patients with gastritis or GC and divided (Table 1).

#### 2.3.2. Sample Preparation (Stimulation with Single Bacteria)

AGS cells were seeded at 5.0 × 10^5^ mL^−1^ in F-12 medium containing 10% FCS and incubated at 37 °C for 24 h to allow adherence to the plate. The medium was subsequently replaced with F-12 medium without FCS, and the cells were incubated at 37 °C for 24 h for synchronization. The medium was then replaced with F-12 medium containing 10% FCS, and the culture supernatants of the nitrate-reducing strains and *H. pylori* (ATCC43504) that were previously cultured in nitrate-supplemented liquid broth for 24 h were added, and the cultures were incubated at 37 °C for 24 h. The F-12 medium was removed to detach cells from the bottom of the plate, and the plate was then washed with phosphate-buffered saline (PBS) and TrypLE™ Express Enzyme (1X; Thermo Fisher Scientific). Subsequently, phenol red (Thermo Fisher Scientific) was added, and the cells were incubated at room temperature for 10 min. The cells were collected, washed three times with PBS, and fixed by incubation in 70% ethanol at 4 °C for 24 h.

#### 2.3.3. Measurement and Analysis

Cell cycle measurements were performed using a CytoFlexS flow cytometer (Beckman Coulter, Brea, CA, USA). Cell cycle analysis was performed using CytExpert Ver. 2.7 analysis software (Beckman Coulter).

### 2.4. Cytokine Measurements

#### 2.4.1. Sample Preparation (Stimulated with Monocultured Bacteria)

The NRB strains and *H. pylori* bacterial suspensions, adjusted to an OD_600_ of 0.5, were added to THP cells (1.0 × 10^6^ mL^−1^), incubated at 37 °C for 6 h, and the culture supernatants were collected.

#### 2.4.2. Measurement

Cytokine production was quantified using an enzyme-linked immunosorbent assay (ELISA). Tumor necrosis factor (TNF)-α was measured using a Human TNF-alpha Un-coated ELISA Kit (Thermo Fisher Scientific), and interleukin (IL)-8, IL-10, IL-12 were measured using CXCL8/IL-8, IL-10, and IL-12 p70 Human DuoSet Kits (R&D Systems, Minneapolis, MN, USA), respectively, according to the manufacturer’s instructions. The absorbance was measured using a MULTISKAN FC instrument (Thermo Fisher Scientific). A colorimetric reagent containing o-phenylenediamine was used as the substrate for IL-8 and IL-12, and the absorbance was measured at 492 nm using the MULTISKAN FC instrument. The measurements were averaged across two or three replicates.

### 2.5. Data Analysis

Statistical significance between groups was determined using Scheffe’s *F* test for multiple group comparisons and Student’s *t* test for two-group comparisons. In both cases, *p* < 0.05 was considered significant.

## 3. Results

### 3.1. Culturing Nitrate-Reducing Bacteria

#### Number of Isolates Other than *H. pylori*

**Figure 1:**
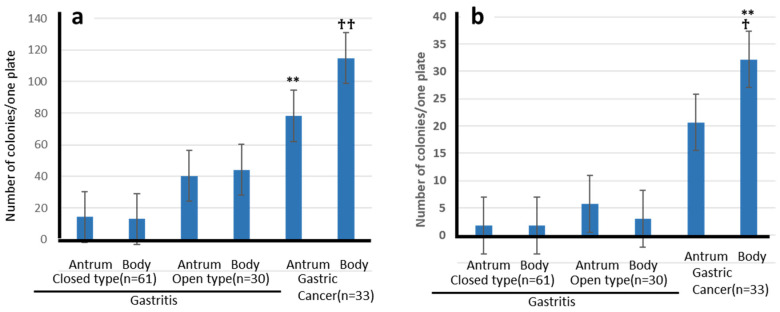
Number of non-*H. pylori* isolates from the stomach. Number of bacteria other than *H. pylori* (**a**); number of Nitrate-reducing bacteria (**b**). The number of colonies are expressed as a mean bar ± SE. ** *p* < 0.01 (compared with closed type), † *p* < 0.05 (compared with open type), †† *p* < 0.01 (compared with open type).

In patients with gastritis, the number of isolates other than *H. pylori* increased with the progression of gastric mucosal atrophy, which also increased with the progression of GC (Figure 1a). There was a significant correlation between the number of other isolates in patients with gastritis and those with early GC was cancer (*p* < 0.01). More bacteria were detected in the antra of patients with gastritis than in the gastric bodies of patients with GC. Similarly, the number of NRB isolates increased with the progression of gastric mucosal atrophy. Moreover, the number of NRB isolates was significantly higher in patients with GC than in those with gastritis (*p* < 0.01, closed type; *p* < 0.05, open type) (Figure 1b). In addition, the rate of NRB isolation increased as atrophy progressed, with open-type gastritis, closed-type gastritis, and GC accounting for 22%, 40%, and 53% of cases, respectively.

### 3.2. Nitrate Reduction Activity of Nitrate-Reducing Bacteria Isolated from Gastritis–Gastric Cancer Patients

#### 3.2.1. Slow-Growing Bacteria

**Figure 2:**
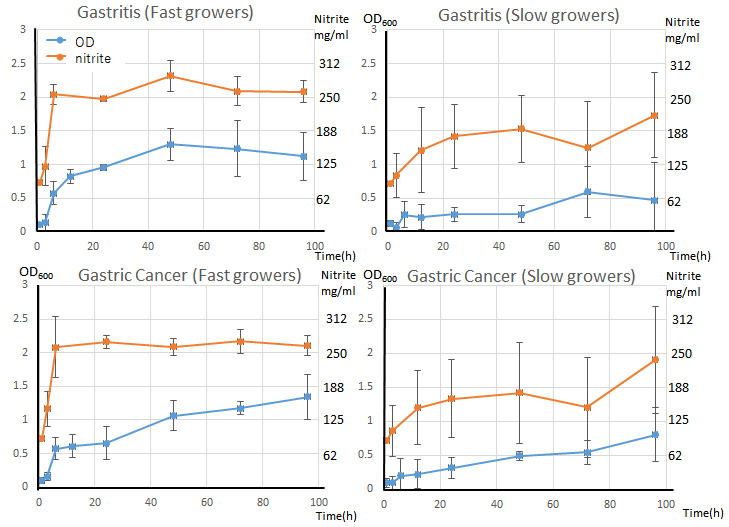
Bacterial growth and nitrite concentration over time. Bacteria from gastritis (fast-growing strains: *n* = 4; slow-growing strains: *n* = 7) and from gastric cancer (fast-growing strains: *n* = 8; slow-growing strains: *n* = 7) were cultured from 3 to 96 h. Data is indicated as mean ± standard error (SE).

Nitrite-concentration and bacterial-growth curves were plotted for each group, separated into gastritis and GC patient samples, and the trends were plotted (Figure 2). Overall, both the nitrite concentration and growth curves tended to increase gradually with time. Most of the bacteria originated from the oral cavity.

#### 3.2.2. Fast-Growing Bacteria

Nitrite-concentration and bacterial-growth curves for fast-growing bacteria are shown in Figure 2. Most bacteria were Enterobacteria of intestinal origin. Overall, slow-growing bacteria tended to increase gradually, whereas fast-growing bacteria showed an early and rapid increase in bacterial count. Subsequently, the nitrite concentration plateaued, whereas the bacterial counts tended to increase gradually throughout.

#### 3.2.3. Maximum Nitrate-Reduction Activity (Vmax)

The maximum nitrite production rates (Vmax) between the two steepest slopes on the nitrite concentration graph was used for comparison. The nitrate reduction activities of the gastritis and GC groups were compared.

**Figure 3:**
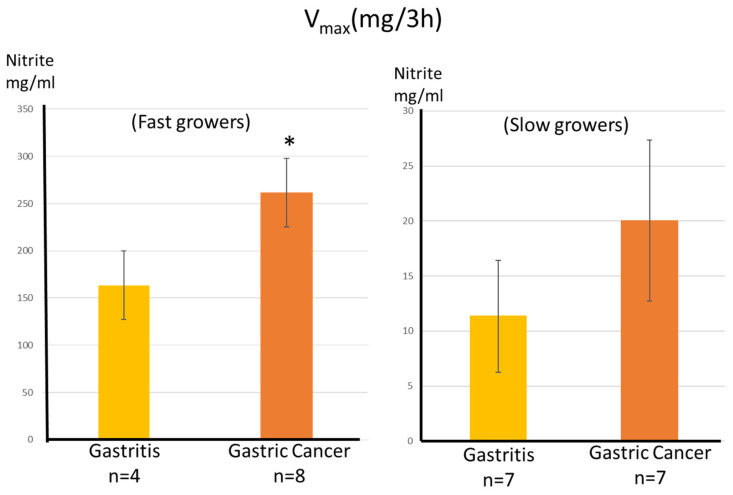
Maximum nitrate-reduction capacity. Comparison of maximum nitrite production after 3 h between the gastritis and gastric cancer strains. Gastric cancer-derived strains tended to have a higher production capacity. The values are expressed as a mean bar ± SE. A significant difference (* *p* < 0.05) was observed in the rapidly growing strains.

For slow-growing bacteria, NRB from patients with GC had a higher nitrate-reducing ability, but no significant differences were observed. By contrast, when the Vmax of fast-growing bacteria was compared, NRB from patients with GC were found to have a significantly higher nitrate-reducing ability (Figure 3).

### 3.3. Effects on Cell Cycle

#### Single Bacterial Stimulation

**Figure 4:**
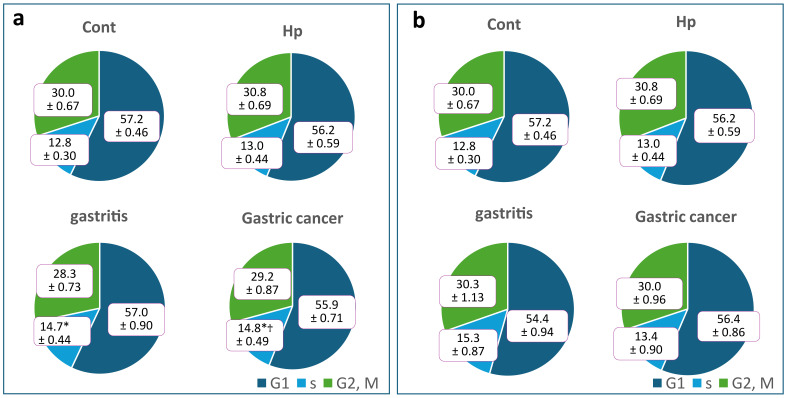
Cell cycle analysis results. Comparison of the effects of NRB strains derived from patients with gastritis and gastric cancer (GC) on cell cycle progression. Values are presented as mean ± SE. All figures are rounded to one decimal place. (**a**) Slow growing bacterial populations (strains from patients with gastritis: *n* = 7; strains from patients with GC: *n* = 7). (**b**) Intestinal fast growing bacterial populations (strains from patients with gastritis: *n* = 4; strains from patients with GC: *n* = 8). (* *p* < 0.05, as compared to control (Cont.); † *p* < 0.05, as compared to *H. pylori* monoculture (Hp)).

**Figure 5:**
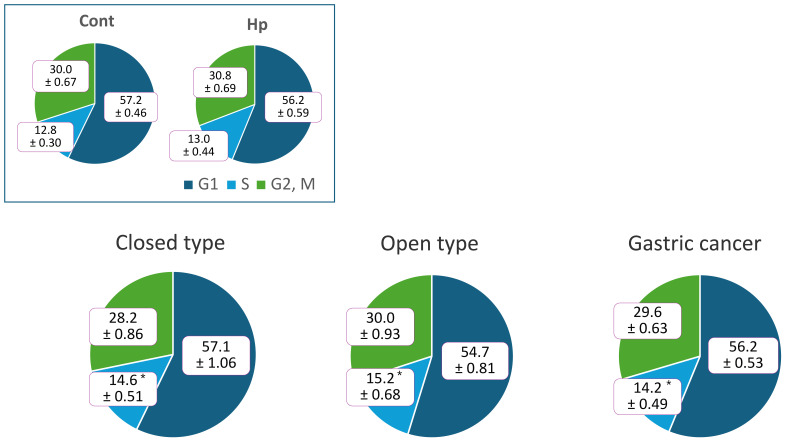
Analysis of cell cycles in different gastric mucosal atrophy levels in patients with gastritis. Comparison of cell cycle progression between closed- (*n* = 6) and open-type (*n* = 5) cell lines derived from patients with gastritis and GC (*n* = 15). Values are presented as mean ± standard error (SE). All figures are rounded to one decimal place (* *p* < 0.05, as compared to Cont.).

We divided strains derived from patients with gastritis and GC into slow growing and fast growing bacterial groups for comparison. When cells were stimulated with slow growing oral bacteria, strains derived from patients with GC tended to progress through the cell cycle, whereas when cells were stimulated with fast growing intestinal bacteria, strains derived from patients with gastritis tended to progress through the cell cycle (Figure 4). S-phase cells in the oral bacterial group were significantly higher in both patients with gastritis and GC compared with the unstimulated controls. Furthermore, strains derived from patients with GC showed significant differences from those derived from patients stimulated with *H. pylori* monoculture (Hp). When we compared strains derived from the three symptom groups (closed-type, open-type, and GC), strains derived from patients with open-type gastritis—a precursor to GC—tended to progress through the cell cycle (Figure 5).

### 3.4. Cytokine Measurement

#### 3.4.1. Cytokine Production upon Stimulation with Nitrate-Reducing Bacteria

*H. pylori* and NRB were separated and compared according to disease stage.

#### 3.4.2. TNF-α

**Figure 6:**
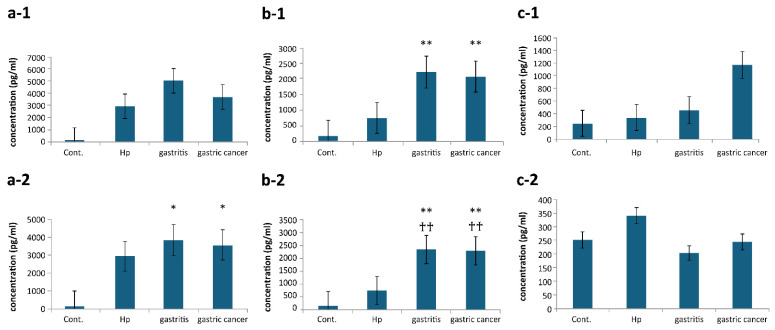
Production amount of cytokine. The levels of TNF-α, IL-8, and IL-10 produced when NRB strains derived from patients with gastritis and GC were treated. (**a**) TNF-α, (**b**) IL-8, and (**c**) IL-10. 1: slow growing bacterial group (strains derived from patients with gastritis: *n* = 7; strains derived from patients with GC: *n* = 7), 2: fast growing bacterial group (strains derived from patients with gastritis: *n* = 4; strains derived from patients with GC: *n* = 8). The values are expressed as a mean bar ± SE. (* *p* < 0.05, as compared to Cont; ** *p* < 0.01, as compared to Cont; †† *p* < 0.01, as compared to Hp).

Higher levels of TNF-α―an indicator of cytotoxicity―was produced by strains in the slow growing bacterial group derived from patients with gastritis than by those derived from patients with GC (Figure 6(a-1)). By contrast, TNF-α production in the fast growing bacterial group of the strains derived from patients with gastritis was slightly higher than those in those of the strains derived from patients with GC. Furthermore, both strains derived from patients with gastritis and GC showed a significant increase (*p* < 0.05) in TNF-α compared to the control (Figure 6(a-2)).

#### 3.4.3. IL-8

Stimulation by NRB in the slow growing bacterial group showed a significant difference (*p* < 0.01) in concentrations of the pro-inflammatory cytokine IL-8 compared to the control group, regardless of the disease stage (Figure 6(b-1)). Similarly, stimulation with NRB in the fast growing bacterial group resulted in a significant difference (*p* < 0.01) compared with the control and Hp groups, regardless of the disease stage (Figure 6(b-2)). TNF-α and IL-8 showed similar trends between the slow growing oral and fast growing intestinal bacterial groups, but the latter tended to show larger differences between the control and Hp groups.

#### 3.4.4. Th1/Th2-Related Cytokines

We measured the production of IL-10 and IL-12, which are involved in cellular and humoral immunity, respectively. IL-10 production increased in the slow growing bacterial group as gastritis progressed to GC (Figure 6(c-1)). By contrast, in the fast growing bacterial group, stimulation with NRB reduced IL-10 production compared with that in the control and Hp groups (Figure 6(c-2)). IL-12 was below the detection limit in all strains, except for two strains of *Rothia mucilaginosa* derived from patients with GC.

## 4. Discussion

Between patients with gastric mucosal atrophy and GC, the number of isolated bacteria (excluding *H. pylori*) and NRB from those with GC was significantly higher. Furthermore, identification of bacterial species revealed that the proportion of NRB, particularly fast growing enterobacteria, increased as gastric mucosal atrophy progressed to GC. This is consistent with genetic analyses of the gastric microbiota of patients with GC and chronic gastritis from a previous study [11], which revealed an increase in Enterobacteriaceae in patients with GC. These findings suggest that NRB, particularly fast growing enterobacteria, are involved in GC progression.

When cell cycle progression was compared between the oral and intestinal bacterial groups within different symptom groups, the results were reversed. This may be due to large individual differences in strains derived from patients with gastritis, as some patients may develop GC in the future. In fact, when strains derived from patients with gastritis were divided into those with closed-type gastritis and those with open-type gastritis and compared with those derived from patients with GC, strains derived from patients with open-type gastritis tended to progress through the cell cycle more rapidly.

Japan has the third-highest GC incidence rate worldwide [3], but its *H. pylori* infection rate is comparable to or lower than those of other Asian regions [1]. It has been reported that fundus-predominant gastritis is more common in *H. pylori*-positive individuals in Japan than in other Asian regions [13]. These findings suggest that advanced fundus-predominant gastritis is a risk factor for GC, particularly in Japan. Our observation of cell cycle progression in open-type gastritis is consistent with reports that fundus-predominant gastritis is a risk factor for GC. Therefore, patients infected with *H. pylori* who also have NRB in their stomachs and advanced atrophic gastritis may be at a particularly high risk of developing GC. Regular health check-ups for these patients may be beneficial for the early detection of GC.

We demonstrated that monoculture stimulation with NRB increased the production of the cytokines TNF-α and IL-8. Previous studies have shown that serum TNF-α levels increase with the severity of gastric lesions [14]; they have also shown that serum TNF-α levels are significantly higher in patients with GC than in healthy controls, and that they tend to increase from healthy controls, through patients with precancerous gastric lesions, to patients with GC [15]. These findings suggest that NRB infections, which promote TNF-α secretion and enhance cytotoxicity, is involved in GC progression.

In particular, IL-8 production was significantly higher in both oral and intestinal bacteria than in the control, and intestinal bacteria showed a significant difference compared with Hp, suggesting that NRB strongly induces acute inflammatory responses. Previous studies have shown that the overexpression and increased serum levels of IL-8 are associated with the progression of gastric and other gastrointestinal cancers and poor prognosis [16,17,18,19], suggesting that NRB is involved in GC progression.

By contrast, IL-10 production was low in the intestinal bacterial group but increased with symptom progression in the oral bacterial group. M2 macrophages promote tumor progression and IL-10 induces M2 macrophage polarization [20]. Therefore, increased IL-10 secretion promotes tumor formation. Furthermore, research has shown that IL-10-expressing cells increase in the peripheral blood supply and tumor tissue of patients with GC as symptoms progress [21]. Therefore, the results of the present study suggest a relationship between slow growing oral bacteria and GC progression.

IL-12 production was detected only in *R. mucilaginosa*, suggesting that *R. mucilaginosa* may be particularly potent in inducing cell-mediated immunity. A study examining gastric microbiota also found that *Rotia* species were more abundant in patients with GC than in those with gastritis, suggesting that *Rothia* species may also be a risk factor for GC [8].

Based on the results of this study, we believe that NRB are involved in GC progression. However, because there were large differences between the strains in cell cycle analysis results and cytokine production levels, we could not confirm whether a specific bacterial species was a risk factor for GC.

## 5. Conclusions

The numbers of non-*H. pylori* bacteria and NRB isolated from patients with gastritis and GC increased with the progression of gastric mucosal atrophy, and both were significantly elevated in patients with GC. Moreover, NRB isolated from GC had high nitrate-reducing activity. Upon stimulation of gastric epithelial cell lines with the isolated NRB and subsequent cell cycle measurements, the bacteria were found to alter the cell cycle. Furthermore, monocytic cell lines induced the production of cytotoxic and pro-inflammatory cytokines. These results suggest that NRB is involved in GC progression. Therefore, examining the state of gastric atrophy and the microbiome composition may be important for managing the risk of developing GC.

## Figures and Tables

**Table 1:** Bacterial strains in this experiments.

Isolated from Patients with	Fast Growth Strain	Slow Growth Strain
Gastritis	*Klebsiella pneumoniae*	*Rothia mucilaginosa*
	*K. variicola*	*R. dentocariosa*
	*Aeromonas hydrophila*	*Actinomyces odontolyticus*
	*E. coli*	*S. salivarius* (2 strains)
		*Gemella sanguinis*
		*Staphylococcus aureus*
Gastric cancer	*E. fergusonii*	*S. koreensis*
	*E. coli* (2 strains)	*S. salivarius*
	*K. pneumoinae* (3 strains)	*S. constellatus subsp*
	*Streptococcus parasanguinis*	*R.mucilaginosa* (2 strains)
	*Serratia marcescens*	*Actinomyces graevenitzii*
		*S. aureus*

## Data Availability

The original contributions presented in this study are included in the article. Further inquiries can be directed to the corresponding author.

## Abbreviations

The following abbreviations are used in this manuscript:

GC	Gastric cancer
NRB	Nitrate-reducing bacteria
ENOC	Endogenous nitroso compound
Vmax	Maximum nitrate-reduction activity
SE	standard error
Hp	*H. pylori* monoculture
Cont.	Control
PBS	Phosphate-buffered saline
